# Clinical Feature Ranking Based on Ensemble Machine Learning Reveals Top Survival Factors for Glioblastoma Multiforme

**DOI:** 10.1007/s41666-023-00138-1

**Published:** 2023-09-20

**Authors:** Gabriel Cerono, Ombretta Melaiu, Davide Chicco

**Affiliations:** 1 Department of Neurology, University of California San Francisco, San Francisco, CA USA; 2 Dipartimento di Biologia, Università di Pisa, Pisa, Italy; 3 Dipartimento di Informatica Sistemistica e Comunicazione, Università di Milano-Bicocca, Milan, Italy; 4 Institute of Health Policy Management and Evaluation, University of Toronto, Toronto, Ontario Canada

**Keywords:** Glioblastoma, Brain tumors, Survival analysis, Cox proportional hazards, Feature ranking, Machine learning

## Abstract

Glioblastoma multiforme (GM) is a malignant tumor of the central nervous system considered to be highly aggressive and often carrying a terrible survival prognosis. An accurate prognosis is therefore pivotal for deciding a good treatment plan for patients. In this context, computational intelligence applied to data of electronic health records (EHRs) of patients diagnosed with this disease can be useful to predict the patients’ survival time. In this study, we evaluated different machine learning models to predict survival time in patients suffering from glioblastoma and further investigated which features were the most predictive for survival time. We applied our computational methods to three different independent open datasets of EHRs of patients with glioblastoma: the Shieh dataset of 84 patients, the Berendsen dataset of 647 patients, and the Lammer dataset of 60 patients. Our survival time prediction techniques obtained concordance index (C-index) = 0.583 in the Shieh dataset, C-index = 0.776 in the Berendsen dataset, and C-index = 0.64 in the Lammer dataset, as best results in each dataset. Since the original studies regarding the three datasets analyzed here did not provide insights about the most predictive clinical features for survival time, we investigated the feature importance among these datasets. To this end, we then utilized Random Survival Forests, which is a decision tree-based algorithm able to model non-linear interaction between different features and might be able to better capture the highly complex clinical and genetic status of these patients. Our discoveries can impact clinical practice, aiding clinicians and patients alike to decide which therapy plan is best suited for their unique clinical status.

## Introduction

Electronic health records (EHRs) are secure, digitized, longitudinal, recollection of general healthcare data from patients. The introduction of EHRs in modern medicine has added many benefits to healthcare system, such as easier access to data for research, standardized terminology and billing codes, clarity, and anonymity, that make them a superior alternative to paper-based patients records.

Analyzing data from EHRs usually present several challenges, such as large volume data, high dimensional, and unstructured data. Traditional statistical tools often have severe limitations in dealing with these challenges, but machine learning (ML) algorithms have the capacity to deal with these data inferring knowledge and information that otherwise would be unnoticed by medical doctors [1–3]. Computational intelligence driven prognostic and diagnostic tools, in fact, provide precise and quantitative data to clinicians who might aid them to take educated decisions and reduce inter clinical variance.

Survival analysis is a type of regression often used in medicine to estimate time to an event, usually death, to better comprehend the relationships with different covariates and the survival time. Survival prediction is often modeled utilizing Cox proportional hazards regression [4], which has the capacity to model right-censored data. Right-censored data can be described as data that are missing either because the patient dropped out of the study or suffered from a competing event death, which make the dataset incomplete. In the last years, machine learning and deep learning survival models have been developed to overcome different limitations of the original Cox proportional hazards models. Random Survival Forests [5], a modification to the well known Random Forests algorithm, and DeepSurv [6], a feed forward neural network which outputs a hazard function, can take on right-censored data to predict survival. These new algorithms, coupled with high quality data from EHRs, have extreme potential to build better prognostic tools and to better characterize the relationship between patients feature and survival time, for any disease, including glioblastoma.

Glioblastoma multiforme (GM) is a tumor arising from the glia, the non-neuronal component of the nervous system that provides support and protection to the neurons. The WHO grading system classifies glioma tumors into different grades, from I to IV, being I the most benign and IV the most malignant. GM is a type IV gliomal tumor, with a poor prognosis [7]; the median survival is 12 months, with less than 5% of patients survive the 5-year mark. GM is the most common primary tumor of the brain, accounting for the big majority of them [8]. GM patients might have different prognostic factors that might affect survival time such as age, chemotherapy, radiotherapy, and tumor resection, but even under the best prognostic ecosystem, GM is still an aggressive disease with poor prospects [7, 9]. Our study is based from data pulled out of electronic health records (EHRs).

A throughout revision of the scientific literature shows that supervised machine learning has the capacity to model different features of GM disease. Closer inspection to the body of evidence shows that most of the work has been done on radiomics, utilizing different deep learning algorithm to extract features from magnetic resonance imaging (MRI). These radiomics features have been utilized for survival prediction [10], overall prognosis [11], and differential diagnosis [12]. The utilization of clinical features to predict survival is more limited; Senders et al. [13] employed EHR to build a calculator to predict survival from a big amount of patients, but their model lacked important clinical features, such as KPS score, and genomic features like MGMT methylation status and isocitrate dehydrogenase 1 (IDH1) mutation.

In the past few years, gene-targeted therapies have been developed for glioblastoma [14], and the genetic landscape of the tumor has become critical in selecting the proper therapy for patients with this disease. Unfortunately, previous studies do not amalgamate under a single model both clinical and genetic variables. These studies were unable to model the inter-play between key gene mutations and clinical features in risk profiling.

We decided to fill this gap in the scientific literature by analyzing multiple datasets of glioblastoma patients that contained both clinical data and biological markers as features. In this study, we aim to integrate multiple data sets that contain clinical and genomics data points and conduct computational analysis to extract the most important features at the moment of predicting overall survival.

## Datasets

We based our work on the analysis of three datasets to improve robustness and increase inductive power across different populations. We studied the data of these three cohorts through an exploratory data analysis (EDA) [15] and noticed that they did not need any preprocessing steps.

### Lammer Dataset

The Lammer dataset [16] contains data from 60 patients who suffered from glioblastoma multiforme; each patient has 7 features, including overall survival in months, that we used as target variable for time to event prediction (Tables 1, 8, and 9). Patients and histological specimen from them were collected both from Klinikum rechts der Isar (TUM) and at the Klinikum Bogenhausen (STKM) in Munich, Germany. The including criteria were patients with GM that received treatment with surgery, radiation therapy, and temozolomide.

### Shieh Dataset

The Shieh dataset [17] has data from 84 patients who suffered from glioblastoma multiforme, recollected from medical records at two Taiwan Hospitals (Tables 2, 6, and 7). The inclusion criteria were older than 20 years old, good performances status, and undergoing radiation therapy. This dataset contains a total of 9 covariates, including overall survival.

### Berendsen Dataset

The Berendsen dataset [18] comes from 347 patients diagnosed with supratentorial glioblastoma between 2005 and 2013 at the University Medical Center of Utrecht, in the Netherlands. The diagnosis was confirmed with histological examination. The survival data was retrieved from hospital records (Tables 3, 4, and 5).

**Table 1:** Meaning of Lammer dataset features. *MGMT*, O-6-methylguanin-DNA methyltransferase

**Feature name**	**Measurement**	**Meaning**
Age	Years	Age of patients
CHSP70	Binary	Cytosolic heat shock protein 70 expression, low = 0; high = 1
MGMT methylation status	Binary	MGMT promoter methylation, cut off point at 8% of methylated promoters
PFS	Months	Progression free survival
Progress	Binary	Tumor progression, yes = 1, no = 0
Sex	Binary	Male = 1; female = 0

**Table 2:** Meaning of Shieh dataset features. *Gy*, gray units of ionizing radiation

**Feature name**	**Measurement**	**Meaning**
Age	Years	Age of patients
Chemo	Binary	Patient received chemotherapy, yes = 1; no = 0
Dose	Gy	Radiation dose
PFS	Months	Progression free survival
Progress	Binary	Tumor progression yes = 1, no = 0
Sex	Binary	Male = 1; female = 0
Surgery	Binary	Patient received surgery. yes = 1; no = 0.
Volume	mL	Radiation volume

### Scientific Results in Previous Studies

The Lammer dataset study [16] highlighted the role of the expression normal cells cytosolic Hsp70 proteins that is identified by the authors as biomarker for progression free survival of patients diagnosed with glioblastoma. Also, the study of Shieh and colleagues [17] used survival analysis to detect the predictive factors for survival of patients with the same disease: age, diagnosis date, and larger radiation volume. The Berendsen dataset study [18], instead, detected the subventricular zone of the brain as an adverse prognostic factor in glioblastoma. All these three studies employed Cox regression techniques for survival analysis. We summarized the main findings of these studies in Table 10.

**Table 3:** Meaning of Berendsen dataset features. *RT*, radio therapy; *KPS*, Karnofsky performance status; *SVZ*, subventricular zone

**Feature name**	**Measurement**	**Meaning**
Adjuvant treatment	Rank	0: none, 1: monotherapy, 2: RT + TMZ
Age	Years	Age of patients
Biopsy debulking	Binary	0: biopsy, 1: resection
KPS	Binary	KPS: 0: KPS < 70, 1: KPS $\ge$ 70
SVZ status	Binary	0: no SVZ contact, 1: SVZ contact

**Table 4:** Quantitative characteristics of the numeric features of the Berendsen dataset. *s.d*. standard deviation

**Numeric feature**	**Median**	**Mean**	**s.d.**	**Range**
Age	63.00	61.45	12.29	[20, 88]
Survival	276	35.39	295.44	[1, 1000]

## Methods

We selected three different models to predict survival: Cox proportional hazards [19], Random Survival Forests [5], and DeepSurv [6]. All three models have the capacity to process right-censored data, which occurs when the survival time is “incomplete” at the limit of the follow-up time and which standard classifier models are not well suited to model. The first algorithm selected is the Cox proportional hazards model, an extensively used linear model, that we employed as a benchmark before introducing more novel machine learning and deep learning algorithms. The Cox model lacks non-linear modeling capabilities, and its hazard function is proportional across time; due to these limitations, the Cox model is under-powered to model true hazard functions. DeepSurv, a modified deep artificial neural network, can easily model these non-linear relationships among different variables. Unfortunately, artificial neural networks are considered black boxes and tend to over fit. At last, we used a Random Survival Forests, a modified random forest that can model a hazard function out of right-censored data.

The three models were imported from the Python package scikit-survival with default parameters, and because the datasets’ dimensions are relatively small, there was no need of further hyper-parameter tuning.

### Cox Proportional Hazards Model

The Cox proportional hazards model is a semi-parametric regression model that focuses on modeling the hazard function [19]. In its essence, the Cox model consists of only two parts, the baseline hazard function that models the risk of event per change of unit of time and the effect of the multiple covariates. In patients suffering from glioblastoma multiforme, there is an associated hazard function that increases over time and different variables, like age, genetic expression, and treatment, that influence this baseline hazard function. The limitations of the Cox proportional hazards model are that assumes that the hazard rate is constant over time and that covariates influence linearly, and proportionally over time, this hazard rate. Cox hazards model is a proven method that has been used in medicine for decades, and it has been the standard for modeling survival data [20] and has the ability to model time to event in a dataset with right censoring. Moreover, the Cox model was used for survival analysis in all the three original studies on the datasets analyzed in this article [16–18].

**Table 5:** Quantitative characteristics of the category features of the Berendsen dataset #: number of patients at the medical checkup. %: percentage of of patients at the medical checkup

**Category feature**	**#**	**%**
SVZ status (0: no)	240	37.09
SVZ status (1: yes)	371	57.34
SVZ status (none: missing)	36	5.56
KPS (0: < 70)	182	28.12
KPS (1: $\ge$ 70)	461	71.25
KPS (none: missing)	4	0.61
Biopsy debulking (1: biopsy)	223	34.47
Biopsy debulking (2: resection)	424	65.53
Adjuvant treatment (0: none)	144	22.25
Adjuvant treatment (1: monotherapy)	162	25.03
Adjuvant treatment (2: RT + TMZ)	223	34.46
Adjuvant treatment (none: missing)	118	18.23
Survived (0: yes)	150	23.18
Survived (1: no)	497	86.82
Total	647	100.00

### Random Survival Forests

Random Forests (RF) have been proven to work great in medicine, as they have great capacity to generalize the data, and at the same time, these trees methods are interpretable, making RF a great fit for clinical medicine. The Random Survival Forests is a regular Random Forests comprised of survival trees with the capacity to handle right-censored survival data and with the particularity that outputs a cumulative hazard function. Similar to CART, survival trees are binary trees grown by recursive splitting following a survival criterion that maximizes survival difference between daughters nodes. Each tree outputs a cumulative hazard function, with an estimated average cumulative hazard function for the ensemble of survival trees [5]. Random Survival Forests because it has have been used in the past to model survival, and have been shown to be useful in identifying key risk factors [21]. Given the great characteristics of Random Survival Forests, we decided to use it as one of the key machine learning algorithms to model survival.

**Table 6:** Quantitative characteristics of the numeric features of the Shieh dataset. *s.d*. standard deviation

**Numeric feature**	**Median**	**Mean**	**s.d.**	**Range**
Age	61.00	58.60	13.37	[21, 84]
Progression free survival	0.74	0.96	0.82	[0.21, 5.11]
Dose	6000	6040	300	[5000, 6660]
Survival	276	35.39	295.44	[1, 1000]
Volume	247.00	300.80	177.54	[56, 817]

### DeepSurv

DeepSurv is a feed-forward neural network with the capacity to work on survival data [6]. An artificial neural network is a computing model composed of nodes which are connected and have a structure similar to brain cells. These neural networks consist of a number of neurons organized in different layers: an input layer, an output layer, and one or more hidden layers. In the case of DeepSurv, the first layer of neurons takes on the patient’s baseline covariates, followed by a sequence of fully connected layers of neurons, ending with the output of a single node, which has a linear activation function that estimates the log-risk function. This model has the capacity to model highly complex and nonlinear interactions between patient’s variables, thus overpowering the original limitations of the Cox proportional hazards methods. DeepSurv has the downfall of being a black box methods, and although some methods have been developed to increase interpret ability in neural networks, these deep learning algorithms are still limited in its use for clinical medicine due to this limitation. Nonetheless, we decided to still include this algorithm in our analysis so we could compare Random Survival Forests to a neural network model (Tables 6, 7, 8, 9 and 10).

**Table 7:** Quantitative characteristics of the category features of the Shieh dataset. #: number of patients at the medical checkup. %: percentage of of patients at the medical checkup

**Category feature**	**#**	**%**
Sex (0: female)	52	61.90
Sex (1: male)	32	38.10
Surgery (0: no)	21	25.00
Surgery (1: yes)	63	75.00
Chemo (0: no)	12	14.29
Chemo (1: yes)	72	85.71
Progress (0: no)	1	1.20
Progress (1: yes)	83	98.80
Survived(0: yes)	3	3.57
Survived(1: no)	81	96.43
Total	84	100.00

**Table 8:** Quantitative characteristics of the numeric features of the Lammer dataset. *s.d*. standard deviation

**Numeric feature**	**Median**	**Mean**	**s.d.**	**Range**
Age	58	56.98	12.08	[20, 78]
Progression free survival	12.5	15.71	11.74	[0.7, 52.4]
Dose	6000	6040	300	[5000, 6660]
Survival	16.4	19.46	13.60	[0.7, 76.1]

**Table 9:** Quantitative characteristics of the category features of the Lammer dataset. #: number of patients at the medical checkup. %: percentage of of patients at the medical checkup

**Category feature**	**#**	**%**
CHSP70 (0: low)	22	36.67
CHSP70 (1: high)	38	63.33
MGMT methylation (0: no)	37	61.67
MGMT methylation (1: yes)	23	38.33
Sex (0: female)	25	41.67
Sex (1: male)	35	58.33
Progress (0: no)	5	8.33
Progress (1: yes)	55	91.67
Survived(0: yes)	24	40.00
Survived(1: no)	36	60.00
Total	60	100.00

**Table 10:** Main scientific findings in the previous studies on the three analyzed original datasets. #*pt*, number of patients; *ML*, machine learning

		**ML feature**	**Survival**	**Main**
**Dataset, year [reference]**	**#pt**	**ranking**	**analysis**	**finding**
Berendsen dataset, 2019 [18]	347	No	Yes	Cytosolic Hsp70 is strongly associated with survival
Lammer dataset, 2019 [16]	60	No	Yes	Age, diagnosis date, and larger radiation volume are strongly associated with survival
Shieh dataset, 2020 [17]	84	No	Yes	Subventricular zone (SVZ) in the brain is associated with death

### Concordance Index 

The Concordance index (C-index) [22] is one of the most popular metric to evaluate survival models [23]; it is similar to the Wilcoxon-Mann–Whitney statistic [24], and it can be interpreted as the ratio of all the pairs whose predicted survival times are correctly ordered for all the pairs that can be ordered. Two pairs can be ordered if a binary event occurs at different times, and it is concordant if the estimated survival function is lower for the subject that experienced the event at an earlier time. The C-index has 1 as an upper bound and 0 as a lower bound, being 1 a perfect prediction accuracy, 0.5 a random predictor, and 0 a perfect inverse predictor. We based the results obtained in our study on the C-index, since it has a clear meaning and is able to capture the temporal aspect of the analysis [23].

### Integrated Brier Score

The Brier score was originally designed for evaluating probabilistic weather forecast [25]. This score is used for evaluating estimators that output a probability for a certain number of events that can be either binary or categorical, taking into consideration that the events are mutually exclusive and that the sum of the predicted probabilities must be equal to one [26]. The Brier score represents the average squared distance between the status of the even, in this case survival status, and the predicted survival probability. In the case of right censoring in survival datasets, the score is adjusted by weighting the squares distances using the inverse probability of censoring weighting technique (IPCW). The Brier score can take values from 0 to 1, being 0 the best possible value and 1 the worst possible. In this work, we used the integrated Brier score (IBS) that outputs a score of the model at all available times in the dataset [27].

### Dataset Split and Feature Ranking

We run the survival modeling of each method 100 times, each time with a random splitting of 70% for training data and 30% for test data. We reported the average score for the 100 runs of the models. The ranking we implemented was based on a recursive feature permutation algorithm, where we permuted each feature once and measured the lost of C-index; we only used Random Survival Forests for the ranking as this known algorithm can be explainable. We also run this algorithm 100 times, with a random splitting each time, saving the loss C-index for each feature and producing a ranking; the higher the loss of C-index, the higher the ranking. After the 100 runs, we merged the rankings utilizing the Borda’s count method, where we summed up each single ranking and divided by the number of loops.

### Biostatistics Univariate Tests

The machine learning methods can inform us about the importance of each variable when all the variables are used together. Since we wanted to detect the relevance of each feature when used alone, we decided to employ some traditional biostatistics approaches to this end. We investigated the relationship between each clinical feature and the survival target. We generated the *p*-values through the Chi-square test [28] and Mann–Whitney *U* test  [29]. These tests, applied to two vectors of real values, return an outcome *p*-value in the [0, 1] range: if there is a statistical correlation between the two vectors, the outcome is close to 0; if there is no statistical correlation between the two vectors, the outcome is close to 1. We consider significant only the results where the *p*-value is lower than 0.005, as suggested by Benjamin et al. [30]. Biostatistics test results can provide alternative information about the relationship between each clinical factor and the survival feature, in addition to the knowledge revealed by machine learning [31].

### Software Packages

We implemented our methods in Python by using the pandas, matplotlib, numpy, scikit-survival, scipy, and sklearn software packages on a Google Colaboratory Jupyter notebook. For the concordance index (C-index) and the Brier score, we utilized the sksurv.metrics library. Our software code is publicly available on GitHub for anyone to use it (Software and data availability).

## Results

In this section, first we explore the results obtained by the different survival analysis methods (Section 4.1); subsequently, we disclose and comment on the results obtained by the ranking method previously outlined (Section 4.2).

### Survival Analysis

**Fig. 1:**
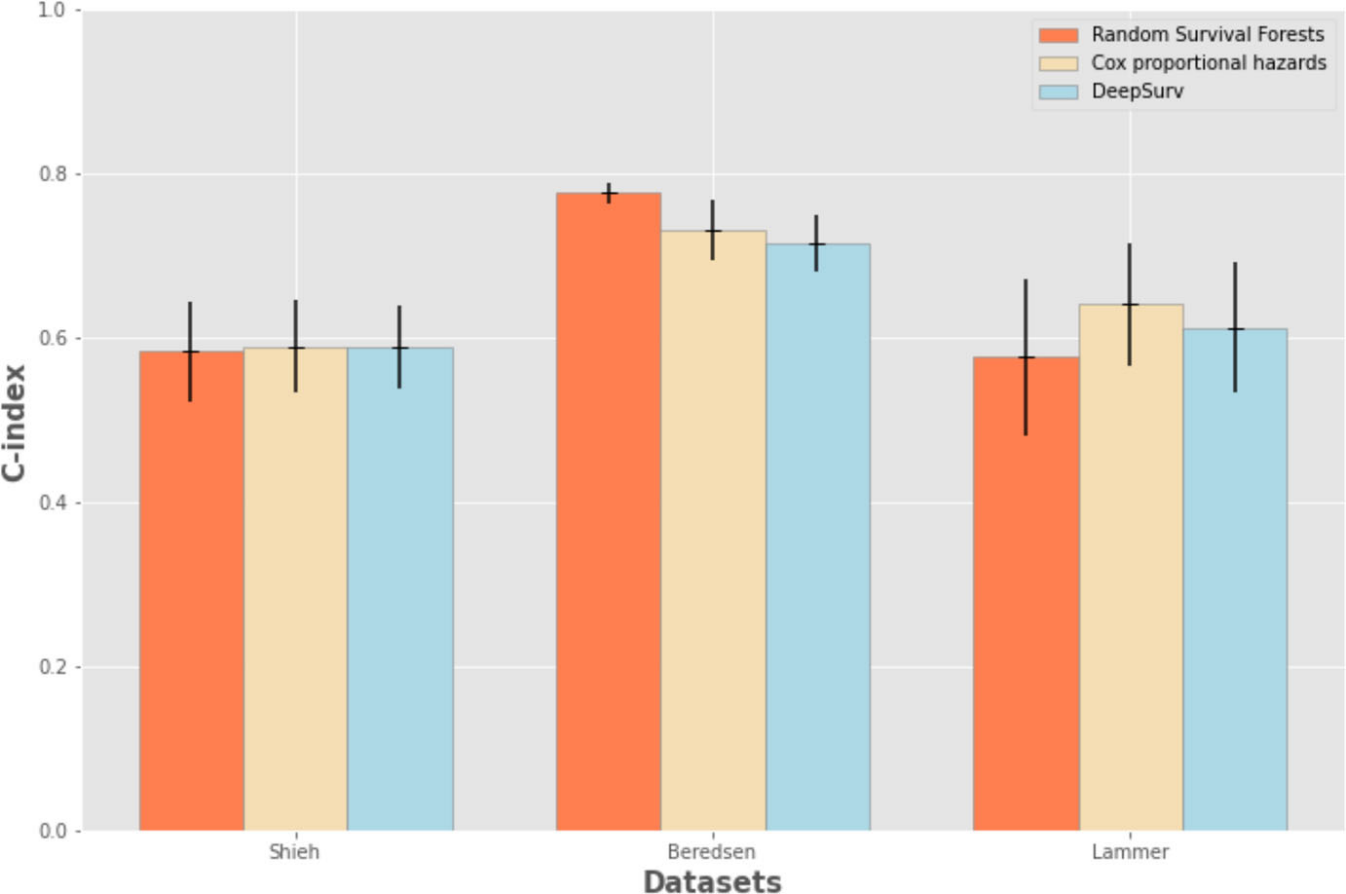
Survival analysis results. Representation of the survival analysis results reported as mean C-index ± the corresponding standard deviations for each method. We reported the complete results measured with other rates in Tables 11, 12, and 13

Among the different algorithms utilized for modeling the survival function, Random Survival Forests outperformed both classical Cox proportional hazards and neural networks (DeepSurv) in the Berendsen dataset, achieved approximately the same results of the two other methods in the Shieh dataset, and was outperformed by DeepSurv in the Lammer dataset (Fig. 1). Random Survival Forests achieved an average C-index of 0.77 in the Berendsen dataset, an average C-index of 0.64 on Lammer dataset, and an average C-index of 0.58 in the Shieh dataset (Fig. 1, Tables 11, 12, and 13).

**Table 11:** Survival regression results on Shieh dataset

**Method**	**C-index**	**IBS**
Random Survival Forests	**0.583 ± 0.061**	0.137 ± 0.025
DeepSurv	*0.589 ± 0.057	0.134 ± 0.028
Cox proportional hazards	**0.588 ± 0.050**	0.143 ± 0.035

Performance of the different survival models evaluated with the C-index and integrated Brier score metrics, expressed in the format “average value ± standard deviation.” The results were acquired from 100 executions, each one had the model trained and evaluated from randomly data selected from the original dataset. The partitioning was 33.3% for the training set, 33.3% for the ranking set, and 33.3% for evaluation set. We reported in blue and with an asterisk (*) the top result for each rate. At the beginning of each execution, we randomly shuffled the dataset instances. *C-index*, concordance index; *IBS*, integrated Brier score. The complete formulas for the scores can be found in the Supplementary Information

**Table 12:** Survival regression results in Berendsen dataset. These results refer to the same abbreviation meanings and execution details of Table 11 caption

**Method**	**C-index**	**IBS**
Random Survival Forests	*0.776 ± 0.013	0.128 ± 0.005
DeepSurv	**0.731 ± 0.036**	0.138 ± 0.012
Cox proportional hazards	**0.715 ± 0.034**	0.147 ± 0.015

**Table 13:** Survival regression results in Lammer dataset. These results refer to the same abbreviation meanings and execution details of Table 11 caption

**method**	**C-index**	**IBS**
Random Survival Forests	**0.576 ± 0.095**	0.184 ± 0.025
DeepSurv	*0.640 ± 0.075	0.161 ± 0.026
Cox proportional hazards	**0.612 ± 0.079**	0.182 ± 0.042

### Ranking of Features Results

After predicting the survival function, we utilized Random Survival Forests and a recursive feature permutation algorithm [32] to rank the features according to its importance in the prediction. We employed Random Survival Forests because it obtained the highest prediction result among the thee methods applied to the three datasets (C-index = 0.776 in the Berednesen dataset, Fig. 1), and it obtained it in the dataset with most patients. In fact, there are data of 647 patients in the Berednesen dataset, while the Shieh dataset contains data of only 85 patients, and the Lammer dataset holds data of only 60 patients. Moreover, Random Survival Forests has been proved to be one of the most effective methods for feature ranking and feature selection in health informatics [5], especially in analyses of electronic health records [33, 34].

In the Lammer dataset, we found that cytosolic heat shock protein 70 expression and MGMT-methylation were the most important factor to predict survival, while age and sex were the least important (Table 14).

The permutation feature importance algorithm found age and dose to be the most important factor in the Shieh dataset (Table 15). Volume of the tumor, radiation dose, and chemotherapy were among the key factors for prediction, while sex and surgery were found to be unimportant.

The same algorithm run on the Berendsen dataset also found chemotherapy to be a key factor to predict survival (Table 16). In contradistinction with the Shieh dataset, biopsy debulking (surgery) was found to be also an important factor. KPS and SVZ status were found on the bottom of the ranking.

**Table 14:** Feature ranking results obtained through Random Survival Forests on Lammer dataset. *s.d*. standard deviation. The computed average Borda score on 100 executions of Random Survival Forests. At each instance of execution, we shuffled the original dataset in 3 sub sets (training, rank, and validation sub sets)

**Rank**	**Feature**	**Average Borda score**	**s.d.**
1	MGMT methylation	1.68	1.00
2	CHSP70	2.54	1.26
3	Progress	3.09	1.02
4	Age	3.81	1.31
5	Sex	3.88	1.09

**Table 15:** Feature ranking results obtained through Random Survival Forests on Shieh dataset. *s.d*. standard deviation. The computed average Borda score on 100 executions of Random Survival Forests. At each instance of execution, we shuffled the original dataset in 3 sub sets (training, rank, and validation sub sets)

**Rank**	**Feature**	**Average Borda score**	**s.d.**
1	Age	2.16	1.81
2	Dose	2.52	1.55
3	Volume	4.40	2.20
4	Chemo	4.41	1.40
5	Status PFS	4.67	1.18
6	Surgery	4.90	1.71
7	Gender	4.92	1.74

**Table 16:** Feature ranking results obtained through Random Survival Forests on Berendsen dataset. *s.d*. standard deviation. The computed average Borda score on 100 executions of Random Survival Forests. At each instance of execution, we shuffled the original dataset in 3 sub sets (training, rank, and validation sub sets)

**Rank**	**Feature**	**Average Borda score**	**s.d.**
1	Adjuvant treatment	1.00	0.00
2	Biopsy debulking	2.42	0.63
3	Age	2.88	0.73
4	SVZ status	3.93	0.72
5	KPS	4.77	0.42

For completion, we also performed this feature ranking step through traditional biostatistics methods and reported the results in Tables S1, S2, and S3. We considered significant only the clinical features obtaining *p*-value lower than 0.005, following the guidelines of Benjamin et al. [30].

## Discussion

As shown in this piece of work, machine learning models often reveal new insights into prognosis prediction. For example, in the Lammer dataset, progress has the smallest *p*-values out of every covariate, for both the Shieh and Lammer datasets, but in our ranking, this variable occupies a rather unimportant place in the ranking, being 3rd out of 5 for the Lammer dataset and 5th out of 7 for the Shieh dataset (Sect. 4.2). Another variable that has a high variability between our machine learning ranking and biostatistics ranking is the KPS, which is statistically significant in our analysis, but it holds the last place in our machine learning ranking. Other variables, like MGMT promoter methylation, volume of the tumor, and radiation, are in sinchrony, being top variables both the statiscal analysis and the machine learning ranking (Sect. 4.2).

The Karnofsky Performance Scale (KPS) score is a widespread “performance” metric used primarily in oncology. Doctors assign a subjective score to patients depending on everyday life functionality. The score goes from 100 to 0, where 100 is great health and 0 is death, for example, 100 means normal without complaints and without evidence of disease, while 40 is disabled and requires special care and assistance. In patients suffering from glioblastoma multiforme, the KPS scale can be used to stratify them into prognostic groups. Patients with lower KPS are usually not assigned to more invasive therapies like surgery or chemotherapy and instead are recommended hospice care (Sect. 4.2).

KPS has been shown to be a good prognostic factor in several oncologic diseases but might not be a good scale to measure functionality in brain diseases [35].

Studies have shown that patients suffering from glioblastoma multiforme with low KPS increase their survival and, most importantly, their KPS score after resection and radiation [36, 37]. Evidence seems to show that KPS score at diagnosis is rather a biased prognostic factor as patients who initially present with poor KPS can rapidly improve after surgery or radiation therapy. Postoperative KPS scores have been shown to have better predictive capabilities than KPS at diagnosis in terms of overall survival in GBM [38]. Close inspection of the Berendsen et al. [18] study shows that they utilized the pre-surgery KPS. We hypothesize that the KPS score got the last rank position in this dataset mainly due to utilizing KPS score before radiation/surgery therapy instead of after. KPS at diagnosis might not be a great prognostic tool in glioblastoma multiforme; instead, doctors might want to utilize KPS after radiation/surgery if they wish for a better prognostic tool.

Age seems to be highly heterogeneous, being one of the top factor in the Shieh dataset, while being uninmportant in the Lammer and Berendsen dataset, and being a plain unimportant variable across all of the datasets in our statistical analysis. We would need to further analyze other datasets to arrive to a meaningful conclusion about this variable.

The importance of molecular markers in GM, not only for prognostic profiling but for management of patients, has gained traction in recent times. One of the most important biological markers at the present time is MGMT promoter methylation. It has been shown that MGMT-hypermethilated tumors have an increase response to alkiylating drugs, as these cells were unable to repair DNA lesions [39]. Although the importance of MGMT methylation for prognostic factor is still somehow controversial [40], we found that this marker was the most important variable at the moment of predicting overall survival for the Lammer dataset. Heat shock protein 70 is a new biological marker that has been discussed in the past as a possible drug target [41], but that had not previous linkage to GM prognosis. This biologic marker came in second place as one of the most important variable for determining prognosis. Although much more research needs to be done before we can conclude any meaningful relationship, these discoveries find that the pursue of new biological markers might be a fruitful endeavor.

## Conclusions

Glioblastoma multiforme is an aggressive tumor with poor prognosis, a mostly incurable cancer with a median survival from diagnosis of only 15 months, with less than 5% of patients surviving past the 5-year mark [42]. The ability to predict time to event in this population is therefore key to offer high quality care to the patients and their families. Medical care of patients suffering from glioblastoma, and other terminal diseases, should have minimal hospitalizations, limited number of interventions, and early hospice care as valuable outcomes at which to aim [43–45].

In this study, we used different computational intelligent methods in 3 different datasets to predict time to event; in all the datasets, our methods had great capacity to predict survival. After confirming that our method worked, we constructed an importance ranking from the features in the datasets.

Our findings might have a direct and an important impact on the management of patients with GM. The KPS is often used as a pivotal factor to decide prognosis and therefore treatment selection  [46]. Unfortunately, patients that present with brain malignicies often suffer from neurologic symptoms like that might lead to an innacurate Karnofsky performance status [47]. Our analysis found that the pre-surgery KPS was one of the worse performing score. This finding adds to the new evidence that postoperative KPS might be a superior predictor score and should be utilized over preoperative KPS [38].

Finally, our overall approach could have a great impact in clinical practice, as the models could be tried in bigger datasets to accurately predict individual predicted survival time in glioblastoma patients.

We were able to address the shortcomings of previous studies by integrating both clinical and genetic features in our datasets and used machine learning models that can integrate non-linear relationships among these features. This valuable information might help terminal patients to decide the treatment and management they deem most valuable at time of diagnosis.

The main asset of the results of our study is the possibility to indicate to medical doctors and physicians a few clinical features on which to focus when reading the medical records of a patient diagnosed with glioblastoma. When visiting a patient diagnosed with this disease and reading their health record, in fact, a medical doctor can pay more attention to the top clinical factors that our methods indicated in the feature rankings to forecast a potential survival time for the patient. Our recommendations about the most predictive clinical features for survival time can therefore have a huge impact in glioblastoma research.

Regarding limitations, we need to report that unfortunately, the three datasets considered have few clinical variables in common, and our study would have been more impactful if they shared more common clinical features. We looked for other datasets of EHRs of patients with glioblastoma having the same clinical features online, but unfortunately, we could not find them.

## Data Availability

Our software code is available under the GNU General Public License v3.0 (GPL 3.0) license at the following web URL: https://github.com/gabrielcerono/GlioblastomaMultiforme The datasets employed in this study are publically available on FigShare under the Attribution 4.0 International (CC BY 4.0) license at the following web URLs: – Lammer dataset: https://figshare.com/articles/dataset/Clinical_data_of_individual_patients_/14201600 – Shieh dataset: https://figshare.com/articles/dataset/S1_Data_-/12312737 – Berendsen dataset: https://figshare.com/articles/dataset/Adverse_prognosis_of_glioblastoma_contacting_the_subventricular_zone_Biological_correlates/9972809?file=17979143

## List of abbreviations

C-index: Concordance index
CHSP70: Cytosolic heat shock protein 70 expression
EDA: Exploratory data analysis
EHR: Electronic health record
GB, GBM, GM: Glioblastoma multiforme
HR: Hazard ratios
IBS: Integrated Brier score
IPCW: Inverse probability of censoring weighting technique
KPS: Karnofsky Performance Scale
ML: Machine learning
MGMT: O-6-methylguanin-DNA methyltransferase
MRI: Magnetic resonance imaging
OS: Overall survival
PFS: Progression free survival
RF: Random Forests
RT: Radio Therapy
s.d.: Standard deviation
SVZ: Sub ventricular zone
